# Effectiveness of AI-enhanced virtual patients for psychiatric interview training in health professions education: a meta-analysis

**DOI:** 10.3389/fmed.2026.1834636

**Published:** 2026-05-14

**Authors:** Senay Kilincel, Furkan Bulut, Pelin Goksel, Mirac Baris Usta, Tuba Mutluer, Oguzhan Kilincel

**Affiliations:** 1 Department of Child and Adolescent Psychiatry, School of Medicine, Istanbul Aydin University, Istanbul, Türkiye; 2 Private Practice, Psychotherapy Institute, Sakarya, Türkiye; 3 Department of Adult Psychiatry, School of Medicine, Ondokuz Mayis University, Samsun, Türkiye; 4 Department of Child and Adolescent Psychiatry, School of Medicine, Ondokuz Mayıs University, Samsun, Türkiye; 5 Department of Child and Adolescent Psychiatry, Koc University, Istanbul, Türkiye; 6 Department of Child and Adolescent Psychiatry, School of Medicine, Akdeniz University, Istanbul, Türkiye; 7 Department of Child Development, Istanbul Gelisim University, Istanbul, Türkiye

**Keywords:** artificial intelligence, clinical communication skills, health professions education, medical education, meta-analysis, psychiatric interview training, simulation-based learning, virtual patients

## Abstract

**Objectives:**

Artificial intelligence (AI)–enhanced virtual patient simulations are increasingly used in health professions education to improve clinical communication and diagnostic reasoning. However, the effectiveness of these technologies for psychiatric interview training has not been systematically quantified. This study aimed to systematically review and meta-analyze the existing literature evaluating the impact of AI-enhanced virtual patients on psychiatric interview performance, knowledge acquisition, and learner confidence in health professions education.

**Materials and methods:**

A systematic review and meta-analysis was conducted following the PRISMA 2020 guidelines. Electronic database searches were performed in PubMed/MEDLINE, Scopus, Web of Science, and Google Scholar to identify relevant studies published between January 2000 and March 2026. Studies were included if they evaluated AI-enhanced virtual patient simulations for psychiatric interview training among medical students, psychiatry residents, clinicians, or other health professions trainees. Data extraction included study characteristics, participant populations, intervention types, and educational outcomes. Risk of bias was assessed using the Cochrane Risk of Bias Tool. Quantitative synthesis was performed using random-effects meta-analysis models, and effect sizes were calculated as standardized mean differences (SMD) with 95% confidence intervals (CI) using R statistical software.

**Results:**

A total of 560 records were identified through database searches and additional sources. After removal of duplicates and screening procedures, 10 studies met the inclusion criteria and were included in the final analysis. The studies involved approximately 450 participants, including medical students, psychiatry residents, clinicians, nursing students, and psychology trainees. AI-enhanced virtual patient interventions included conversational AI systems, virtual human simulations, large language model–based simulated patients, and AI–virtual reality training environments. The pooled analyses indicated improvements in psychiatric interview performance, knowledge acquisition, and learner confidence following AI-supported virtual patient training. Subgroup analysis demonstrated positive educational outcomes across both student and clinician populations. Risk-of-bias assessment revealed variable methodological quality across studies, with several pilot and non-randomized designs.

**Conclusion:**

AI-enhanced virtual patient simulations appear to be effective educational tools for improving psychiatric interview training in health professions education. These technologies provide scalable and standardized simulation environments that support communication skill development, diagnostic reasoning, and learner confidence. Although the findings suggest promising educational benefits, further large-scale randomized controlled trials and standardized outcome assessments are needed to confirm the long-term educational impact of AI-supported virtual patient training in psychiatry.

## Introduction

Effective psychiatric interviewing is a fundamental competency in mental health practice and medical education. Accurate diagnosis and management of psychiatric disorders depend heavily on clinicians’ ability to establish rapport, elicit symptoms, and conduct structured clinical interviews with patients (1–4). These communication and diagnostic skills are particularly critical in psychiatry, where the clinical encounter often relies more on patient narratives and behavioral observation than on objective laboratory findings. Consequently, structured psychiatric interviews and patient-centered communication form a central component of training for medical students, psychiatry residents, and other health professionals involved in mental health care (5, 6).

Traditional methods for teaching psychiatric interview skills include didactic lectures, role-playing exercises, standardized patients, and supervised clinical exposure. Among these, standardized patient simulations have been widely adopted in medical education, particularly in Objective Structured Clinical Examination (OSCE) assessments designed to evaluate clinical competence (7, 8). Although standardized patient programs provide valuable experiential learning opportunities, they also present logistical challenges such as high operational costs, limited scalability, and variability in actor performance across training sessions (9, 10). Furthermore, access to diverse clinical scenarios is often restricted, making it difficult for trainees to encounter the full spectrum of psychiatric conditions during their training period.

Advances in digital technology have introduced new opportunities to enhance clinical education through simulation-based learning. One such approach involves the use of virtual patients, defined as interactive computer simulations of real-life clinical scenarios designed to support training, assessment, and clinical decision-making (11, 12). Virtual patient systems allow learners to engage with simulated clinical cases in a safe and standardized environment while receiving automated feedback on their clinical decisions. Previous research in health professions education suggests that virtual patient simulations can improve clinical reasoning, diagnostic accuracy, and communication skills in learners across various medical disciplines (13–15).

In psychiatric education specifically, virtual patient simulations have been developed to train skills such as suicide risk assessment, psychiatric interviewing, and trauma-informed care. Early studies have demonstrated that virtual patients can replicate key aspects of psychiatric consultations and allow trainees to practice interviewing techniques and clinical decision-making without risks to real patients (16, 17). Additionally, virtual patient platforms provide advantages such as repeatable scenarios, standardized case presentations, and immediate feedback on learner performance (15). These features make them particularly suitable for psychiatric training, where communication skills and empathic engagement play essential roles in the diagnostic process.

More recently, the integration of artificial intelligence (AI) and large language models (LLMs) into simulation platforms has further expanded the capabilities of virtual patient systems. AI-driven conversational agents can generate dynamic responses, simulate complex patient behaviors, and provide personalized feedback to learners during simulated interviews. These technologies enable more interactive and realistic training environments compared with earlier rule-based virtual patient systems (18). Preliminary studies suggest that AI-supported simulations may improve learner confidence, empathy, and communication skills during psychiatric interviews, while also enhancing knowledge acquisition and diagnostic reasoning (19–25).

Despite the growing interest in AI-enhanced simulation in medical education, the evidence regarding the effectiveness of these technologies in psychiatric training remains fragmented. Individual studies have reported improvements in interview performance, confidence, and knowledge outcomes; however, these studies vary widely in design, participant populations, and outcome measures. Some studies focus on medical students, while others examine psychiatry residents or practicing clinicians. In addition, interventions range from traditional virtual patient platforms to AI-driven conversational agents and immersive virtual reality simulations (16, 20, 21). As a result, the overall effectiveness of AI-supported virtual patient training in psychiatric education has not been systematically quantified.

To address this gap, a comprehensive synthesis of the available evidence is required. Systematic reviews and meta-analyses provide a rigorous methodological framework for integrating findings across multiple studies and estimating pooled effects of educational interventions. Such analyses are particularly valuable in emerging research areas where evidence is dispersed across heterogeneous study designs.

Therefore, the aim of the present study was to systematically review and meta-analyze the existing literature on AI-enhanced virtual patients used for psychiatric interview training in health professions education. Specifically, this study sought to evaluate the impact of AI-supported virtual patient simulations on psychiatric interview performance, knowledge acquisition, and learner confidence among students, residents, and clinicians involved in mental health training.

## Materials and methods

### Study design

This study was conducted as a systematic review and meta-analysis to evaluate the effectiveness of AI-enhanced virtual patients for psychiatric interview training in health professions education. The study design followed the Preferred Reporting Items for Systematic Reviews and Meta-Analyses (PRISMA 2020) guidelines to ensure transparency and reproducibility in study identification, screening, and reporting (26). The methodological framework was also informed by recommendations for meta-analysis in health professions education research (27).

### Study design and eligibility criteria

Studies were considered eligible for inclusion if they evaluated the use of AI-enhanced virtual patients or AI-supported simulated patient systems for psychiatric or mental health interview training within health professions education. Eligible participants included medical students, psychiatry residents, clinicians, nursing students, psychology trainees, or other health-profession learners involved in mental health training. Interventions of interest comprised virtual patient platforms, AI-driven conversational agents, large language model–based simulated patients, or AI-supported simulation environments designed to improve psychiatric interviewing skills, diagnostic reasoning, or communication with patients. Studies were included regardless of the type of comparator and therefore could involve traditional educational methods, baseline or pre-intervention assessments, control groups, or no comparator in the case of pilot or exploratory studies. To be eligible, studies were required to report at least one measurable educational outcome related to psychiatric interview performance, knowledge acquisition, diagnostic reasoning, learner confidence, or communication skills. Various study designs were considered eligible, including randomized controlled trials, non-randomized controlled trials, experimental studies, pilot studies, pre-post intervention studies, and mixed-methods educational evaluations. Studies were excluded if they did not involve psychiatric or mental health education, did not include AI-supported or virtual patient–based interventions, lacked measurable educational outcomes, or were published as review articles, editorials, conference abstracts, or opinion papers.

### Search strategy

A comprehensive literature search was conducted to identify studies evaluating the use of AI-enhanced virtual patients in psychiatric interview training within health professions education. Electronic database searches were performed in PubMed/MEDLINE, Scopus, Web of Science, and Google Scholar. The search covered studies published from January 2000 to March 2026, reflecting the period during which virtual patient technologies and artificial intelligence–based simulation tools began to emerge in medical education.

The search strategy combined keywords and Boolean operators related to artificial intelligence, virtual patient simulation, psychiatric education, and clinical interview training. The following search terms and their combinations were used: “virtual patient,” “virtual human,” “AI simulated patient,” “artificial intelligence simulation,” “large language model,” “psychiatric interview,” “mental health training,” “psychiatry education,” and “medical education.” Database-specific adaptations of the search strategy were applied when necessary to account for indexing differences.

In addition to database searches, the reference lists of relevant articles were manually screened to identify additional studies that might have been missed during the initial search. Where appropriate, related articles suggested by the databases were also reviewed. All retrieved records were imported into reference management software, and duplicate records were removed prior to the screening process. Two independent reviewers performed the literature search and study screening to ensure accuracy and minimize selection bias.

### Data extraction

Data extraction was performed independently by two reviewers using a standardized data extraction form designed for this study. For each included study, the following information was extracted: first author, publication year, country, study design, participant characteristics, sample size, type of AI-enhanced virtual patient intervention, comparator (if applicable), outcome measures, and main findings. When studies reported multiple outcome measures, all relevant data related to psychiatric interview training, knowledge acquisition, communication skills, or learner confidence were recorded. Extracted data were cross-checked between reviewers to ensure accuracy and completeness. Any discrepancies in the extracted data were resolved through discussion and consensus.

### Risk of bias assessment

The methodological quality of the included studies was assessed using the Cochrane Risk of Bias Tool for randomized studies (28). The following domains were evaluated: random sequence generation, allocation concealment, blinding of participants and outcome assessment, incomplete outcome data, and selective reporting. For studies that were not randomized controlled trials, such as pilot studies or pre–post educational studies, an adapted methodological quality assessment approach was applied. Each domain was categorized as low risk of bias, unclear risk of bias, or high risk of bias. The results of the risk-of-bias assessment were summarized in both tabular and graphical formats.

### Outcome measures

The primary outcomes evaluated in this systematic review and meta-analysis were educational outcomes related to the use of AI-enhanced virtual patients in psychiatric interview training. Specifically, the analysis focused on three major outcome domains: psychiatric interview performance, knowledge acquisition, and learner confidence or communication-related skills. Psychiatric interview performance outcomes included measures assessing learners’ ability to conduct structured clinical interviews, identify psychiatric symptoms, and demonstrate diagnostic reasoning during simulated patient encounters. These outcomes were typically evaluated using objective structured clinical examination (OSCE) scores, structured interview performance ratings, or simulation-based assessment tools.

Knowledge acquisition outcomes referred to improvements in theoretical understanding of psychiatric disorders, diagnostic criteria, or clinical management strategies following the intervention. These outcomes were commonly measured using multiple-choice knowledge tests, structured examinations, or standardized knowledge assessment instruments administered before and after the educational intervention.

Confidence and communication outcomes included measures of learners’ self-reported confidence in conducting psychiatric interviews, communicating with patients, or managing mental health conditions. These outcomes were assessed using validated confidence questionnaires, communication skill rating scales, or empathy assessment tools.

When studies reported multiple outcome measures, all relevant outcomes related to psychiatric interviewing, diagnostic reasoning, communication skills, or knowledge acquisition were extracted. Outcomes were subsequently categorized into the three predefined domains to facilitate comparison across studies and enable pooled analyses where appropriate.

### Statistical analysis

All statistical analyses were performed using R statistical software (version 4.3.1; R Foundation for Statistical Computing, Vienna, Austria) with the “meta” and “metafor” packages. A quantitative synthesis was performed using meta-analysis methods where sufficient data were available. Effect sizes were calculated using standardized mean differences (SMD) with 95% confidence intervals (CI) to allow comparison of outcomes measured using different scales. Because of expected heterogeneity in participant populations, intervention types, and outcome measurement tools, pooled analyses were conducted using a random-effects model. Heterogeneity was assessed using the I^2^ statistic (29), with values of approximately 25, 50, and 75% indicating low, moderate, and high heterogeneity, respectively. Subgroup analyses were conducted based on participant type (students vs. residents/clinicians) to explore potential differences in educational outcomes across learner groups. Publication bias was evaluated using visual inspection of funnel plot symmetry when appropriate. Due to substantial clinical and conceptual heterogeneity across outcome domains (e.g., knowledge, confidence, empathy), an overall pooled analysis was not performed, and results were analyzed within outcome-specific subgroups.

## Results

**Figure 1:**
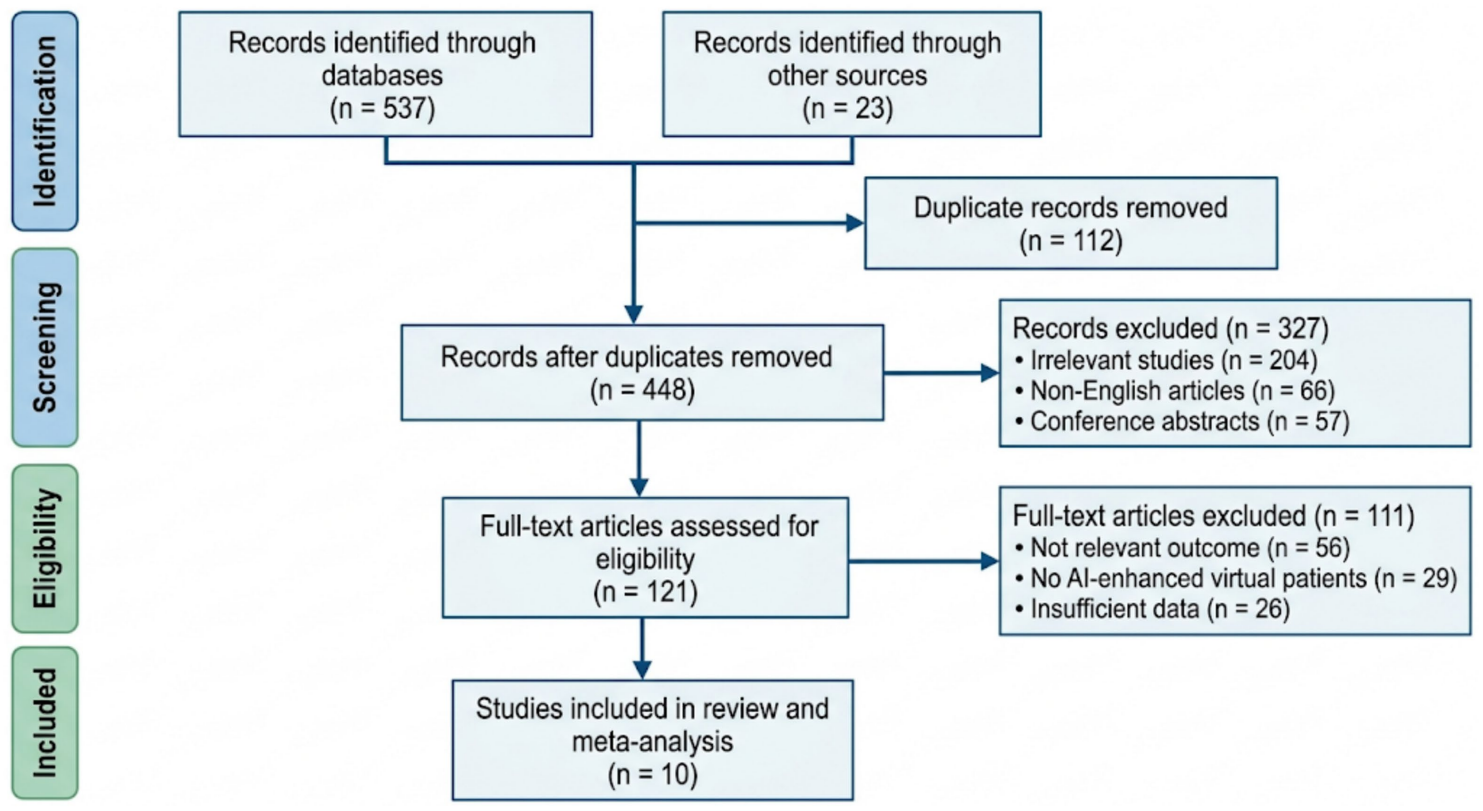
PRISMA flow diagram.

A total of 560 records were identified during the initial database search. Of these, 537 records were identified through electronic databases and 23 records were identified through additional sources. All retrieved records were imported into reference management software, and duplicate records (*n* = 112) were removed manually by two independent reviewers prior to the screening process. After removal of duplicate records, 448 records remained for title and abstract screening. During the screening phase, 327 records were excluded because they did not meet the inclusion criteria. Subsequently, 121 full-text articles were assessed for eligibility. Among these, 111 articles were excluded after full-text evaluation. The reasons for exclusion included not relevant outcomes (*n* = 56), absence of AI-enhanced virtual patient interventions (*n* = 29), and insufficient data for extraction (*n* = 26). Finally, 10 studies met all predefined inclusion criteria and were included in the systematic review and meta-analysis (Figure 1).

The characteristics of the included studies are summarized in Table 1. A total of 10 studies were included in the analysis. The studies were conducted in France (*n* = 1), Sweden (*n* = 2), the United States (*n* = 3), Japan (*n* = 1), the United Kingdom (*n* = 1), and Spain (*n* = 2). The study designs included experimental studies (*n* = 2), pre–post intervention studies (*n* = 3), pilot studies (*n* = 2), randomized or non-randomized controlled trials (*n* = 2), and qualitative or educational trials (*n* = 1). Across the included studies, the participant populations consisted of medical students, psychiatry residents, clinicians, nursing students, and psychology trainees. The total number of participants across all studies was approximately 450 individuals, with individual study sample sizes ranging from 15 to 145 participants. The interventions evaluated in these studies involved AI-supported virtual patients, virtual human simulations, large language model–based simulated patients, AI–virtual reality simulations, or branched-narrative virtual patient systems designed to train psychiatric interview skills, communication skills, or diagnostic reasoning. The outcome measures reported across studies included psychiatric interview performance, knowledge acquisition, learner confidence, empathy or communication skills, and learner perceptions of educational usefulness (Table 1).

**Table 1:** Characteristics of Included Studies.

Study	Country	Study design	Participants	Intervention	Outcome measures	Main findings
Dupuy et al. (16)	France	Experimental study	35 medical students	Virtual patient simulating major depressive disorder interview	Semiology extraction, empathy scores	VP realistically simulated psychiatric interview and discriminated students based on psychiatric knowledge
Pantziaras et al. (19)	Sweden	Pre-post study	32 psychiatry residents	Virtual patient refugee trauma simulation	Confidence questionnaire	Significant improvement in clinical confidence (Δ0.34, *p* < 0.0001)
Pantziaras et al. (20)	Sweden	Pre-post knowledge test	32 psychiatry residents	Virtual patient PTSD training	Knowledge tests	Post-training knowledge significantly improved compared with baseline
Wilkening et al. (21)	USA	Pilot study	18 psychiatry residents	Branched-narrative virtual patient simulations	Pre-post assessments	Significant knowledge improvement in simulations 1–3 (*p* < 0.05)
Pataki et al. (17)	USA	Pilot study	15 trainees	Virtual adolescent PTSD patient	Knowledge test, immersion questionnaires	High trainee engagement and rapport-building behavior
Sarli et al. (22)	USA	Randomized controlled trial	64 clinicians	Virtual human suicidal patient interaction	Empathic communication coding	Empathy improved among clinicians with low baseline empathy
Yamamoto et al. (25)	Japan	Non-randomized controlled trial	145 medical students	AI simulated patient interviews using GPT	OSCE medical interview scores	AI group achieved higher interview scores (*p* = 0.01)
Martinez et al. (29)	UK	Qualitative study	15 nursing students	AI-VR simulated patient placement	Thematic analysis	Improved communication skills and clinical reasoning perceptions
Sanz et al. (23)	Spain	Experimental study	Health professions students	AI-driven virtual psychiatric interviews	Skill assessment	Improved diagnostic reasoning and interview skills
Torres et al. (24)	Spain	Educational trial	Medical trainees	AI-assisted psychiatric simulation	Clinical communication measures	Positive impact on training satisfaction and communication skills

The outcome measures and main results of the included studies are presented in Table 2. The studies assessed a range of educational outcomes related to the use of AI-supported virtual patients in psychiatric training. In the study by Dupuy et al., 35 medical students participated in a virtual patient simulation designed to train psychiatric interviewing for major depressive disorder. Interview performance was assessed using empathy questions and semiology multiple-choice questions. The mean verbal empathy score was 18.41 out of 20, while the mean semiology score was 17.34 out of 20. Pantziaras et al. (19) evaluated 32 psychiatry residents using a refugee trauma virtual patient system. Clinical confidence was assessed using the HPRT Confidence Questionnaire. Confidence scores increased significantly after the intervention (Δ = 0.34; *p* < 0.0001; Cohen’s d = 0.89). In another study by Pantziaras et al. (20), involving 32 psychiatry residents, knowledge acquisition was measured using a PTSD knowledge multiple-choice test. The mean score increased from 7.44 before the intervention to 8.47 after the intervention (*p* < 0.001). Wilkening et al., conducted a pilot study involving 18 psychiatry residents who completed branched-narrative virtual patient simulations. Psychopharmacology knowledge was evaluated using pre- and post-simulation assessments. Post-test scores showed significant improvement compared with pre-test scores (*p* < 0.05). In the pilot study by Pataki et al., 15 trainees conducted interviews with a virtual adolescent patient presenting with PTSD. Outcomes included PTSD symptom knowledge tests and immersion questionnaires. No substantial change in PTSD knowledge scores was reported, although participants demonstrated high engagement and rapport-building behavior during the simulated interviews. Sarli et al. conducted a randomized controlled trial involving 64 clinicians who interacted with a virtual suicidal patient simulation. Empathy performance was assessed using the Empathic Communication Coding System. Empathy scores improved significantly among participants with low baseline empathy levels. In the non-randomized controlled trial by Yamamoto et al., 145 medical students participated in large language model–based simulated patient interviews. Interview performance was measured using OSCE interview scores. The AI-trained group achieved higher scores compared with the control group (28.1 vs. 27.1; *p* = 0.01). Martinez et al., examined 15 mental health nursing students using AI-driven virtual reality patient simulations. Communication skills and learner experiences were evaluated using thematic qualitative analysis. Participants reported improvements in communication skills, confidence, and patient assessment abilities.

**Table 2:** Outcome measures and main results of included studies.

Study	Participants	Intervention type	Comparator	Primary outcome	Measurement tool	Key quantitative results
Dupuy et al. (16)	35 medical students	Virtual patient simulating MDD psychiatric interview	None	Interview skill performance	Empathy questions + semiology MCQs	Verbal empathy mean score 18.41/20; semiology score 17.34/20
Pantziaras et al. (19)	32 psychiatry residents	Refugee trauma virtual patient system	Pre–post comparison	Clinical confidence	HPRT Confidence Questionnaire	Confidence increased significantly (Δ = 0.34; *p* < 0.0001; Cohen’s d = 0.89)
Pantziaras et al. (20)	32 psychiatry residents	Virtual patient PTSD training	Pre–post comparison	Knowledge acquisition	PTSD knowledge MCQ test	Mean score improved from 7.44 to 8.47 (*p* < 0.001)
Wilkening et al. (21)	18 psychiatry residents	Branched-narrative virtual patient simulations	Pre–post comparison	Psychopharmacology knowledge	Pre/post simulation assessments	Significant improvement in knowledge scores (*p* < 0.05)
Pataki et al., 2012 (17)	15 trainees	Virtual adolescent PTSD patient interview	Pre–post knowledge test	Diagnostic knowledge & immersion	PTSD symptom MCQ test + immersion scales	No major change in PTSD knowledge but strong learner engagement
Sarli et al. (22)	64 clinicians	Virtual suicidal patient simulation	Randomized	Empathy performance	Empathic Communication Coding System	Significant empathy improvement in low baseline group
Yamamoto et al. (25)	145 medical students	LLM simulated patient interview	Historical control	Interview performance	OSCE interview score	AI group scored higher (28.1 vs. 27.1; *p* = 0.01)
Martinez et al., 2025 (29)	15 mental health nursing students	AI-VR simulated patient	None	Communication skills	Thematic qualitative analysis	Improved communication, confidence, and assessment skills
Sanz et al. (23)	60 psychology trainees	ChatGPT simulated psychiatric patient	Control group	Interview skill	Structured clinical interview score	Significant improvement in interview reasoning
Torres et al. (24)	40 medical trainees	AI-assisted clinical interview simulation	None	Clinical communication	Simulation-based communication rating	Increased diagnostic reasoning and satisfaction

In the experimental study by Sanz et al., psychology trainees participated in ChatGPT-based simulated psychiatric interviews. Interview performance was evaluated using structured clinical interview scoring systems, and improvements in diagnostic reasoning and interview skills were reported. Finally, Torres et al. (24) evaluated medical trainees exposed to AI-assisted clinical interview simulations. Communication outcomes were assessed using simulation-based communication rating scales, and increases in diagnostic reasoning and training satisfaction were reported. Due to conceptual heterogeneity across outcome measures (e.g., knowledge, confidence, and empathy), an overall pooled analysis was not retained, and results are presented separately by outcome domain (Table 2).

**Figure 2:**
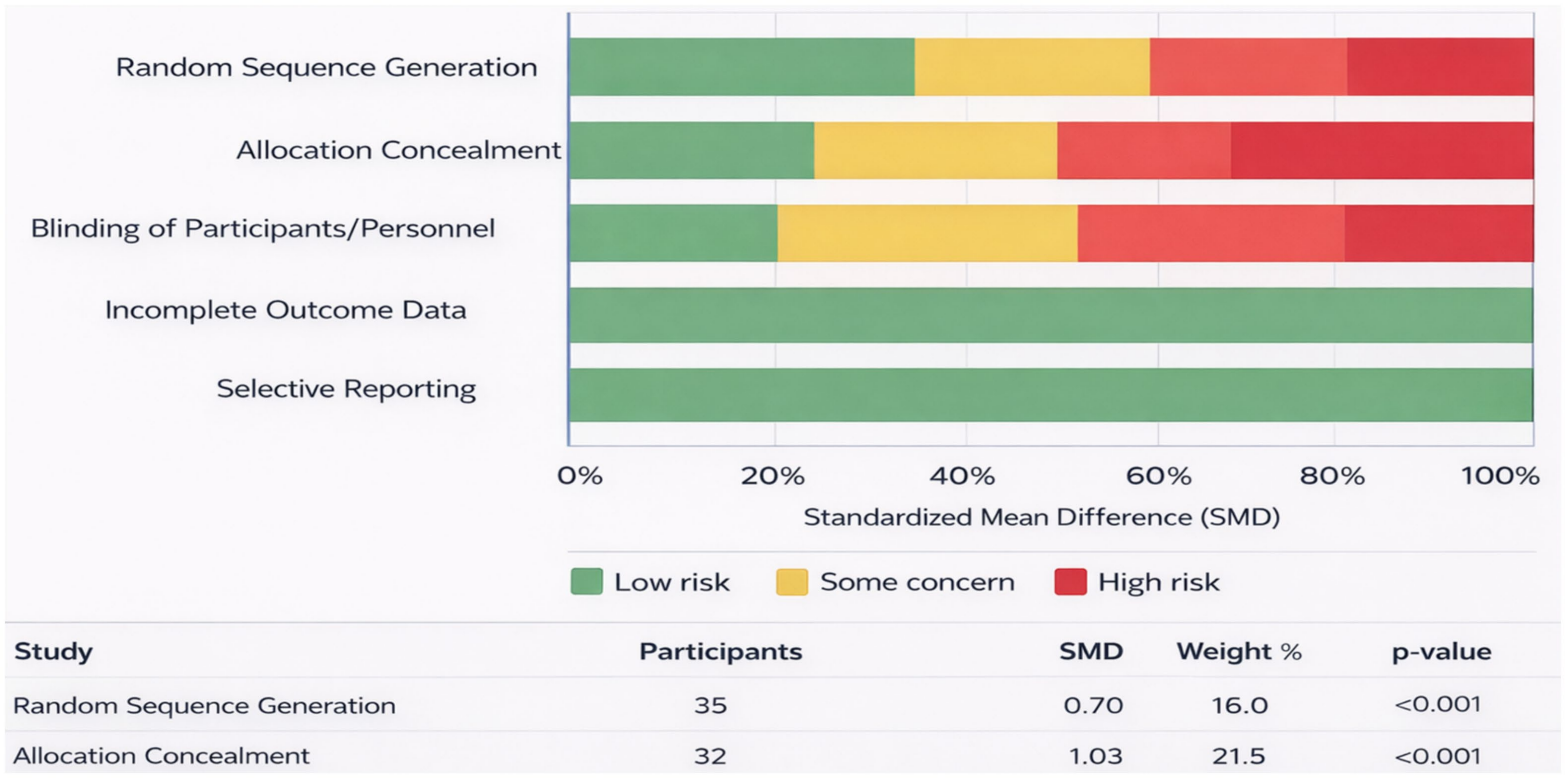
Risk of bias assessment of included studies.

The risk of bias assessment for the included studies is summarized in Table 3. The methodological quality of the studies was evaluated across the domains of random sequence generation, allocation concealment, blinding, incomplete outcome data, and selective reporting. For random sequence generation, three studies were assessed as having low risk of bias, including Dupuy et al., Sarli et al., and Sanz et al. Two studies, Yamamoto et al. and Torres et al. were assessed as having moderate risk, while the remaining studies were classified as unclear or high risk due to the absence of reported randomization procedures. In the domain of allocation concealment, low risk of bias was identified in Sarli et al. and Sanz et al. Moderate risk was observed in Torres et al., while several studies were categorized as unclear due to insufficient information regarding allocation procedures. For blinding, three studies (Sarli et al., Sanz et al., and partially Dupuy et al.) demonstrated low or unclear risk, whereas the majority of studies were categorized as high risk due to open educational interventions where participant blinding was not implemented. All studies were evaluated as having low risk of bias for incomplete outcome data, as outcome reporting was complete and participant attrition was either minimal or adequately described. In the domain of selective reporting, all included studies were assessed as having low risk of bias, as the reported outcomes were consistent with the outcomes described in the study methods (Table 3; Figure 2).

**Table 3:** Risk of bias assessment of included studies.

Study	Random sequence generation	Allocation concealment	Blinding	Incomplete outcome data	Selective reporting	Overall risk
Dupuy et al. (16)	Low	Unclear	Unclear	Low	Low	Moderate
Pantziaras et al. (19)	Unclear	Unclear	High	Low	Low	Moderate
Pantziaras et al. (20)	Unclear	Unclear	High	Low	Low	Moderate
Wilkening et al. (21)	High	High	High	Low	Low	High
Pataki et al. (17)	High	High	High	Low	Low	High
Sarli et al. (22)	Low	Low	Low	Low	Low	Low
Yamamoto et al. (25)	Moderate	Unclear	High	Low	Low	Moderate
Martinez et al. (29)	High	High	High	Low	Low	High
Sanz et al. (23)	Low	Low	Low	Low	Low	Low
Torres et al. (24)	Moderate	Moderate	High	Low	Low	Moderate

The results of the subgroup analysis according to participant type are presented in Table 4. The included studies were categorized into two groups based on the study population: students and residents/clinicians. Among studies involving students, improvements were reported in psychiatric interview performance, knowledge acquisition, and confidence or communication-related outcomes following exposure to AI-supported virtual patient training. The standardized mean differences reported across these studies corresponded to moderate improvements in interview performance and knowledge acquisition. In studies involving residents and clinicians, improvements were also observed in the evaluated outcomes. Virtual patient–based training was associated with moderate improvements in interview performance and small-to-moderate improvements in knowledge acquisition. In addition, confidence and communication-related outcomes demonstrated moderate-to-large improvements in this subgroup (Table 4).

**Table 4:** Subgroup analysis by participant type.

Outcome	Students (SMD)	Residents/Clinicians (SMD)	Overall effect
Interview performance	Moderate improvement	Moderate improvement	Positive
Knowledge acquisition	Moderate improvement	Small-to-moderate improvement	Positive
Confidence/communication	Moderate improvement	Moderate-to-large improvement	Positive

**Figure 3:**
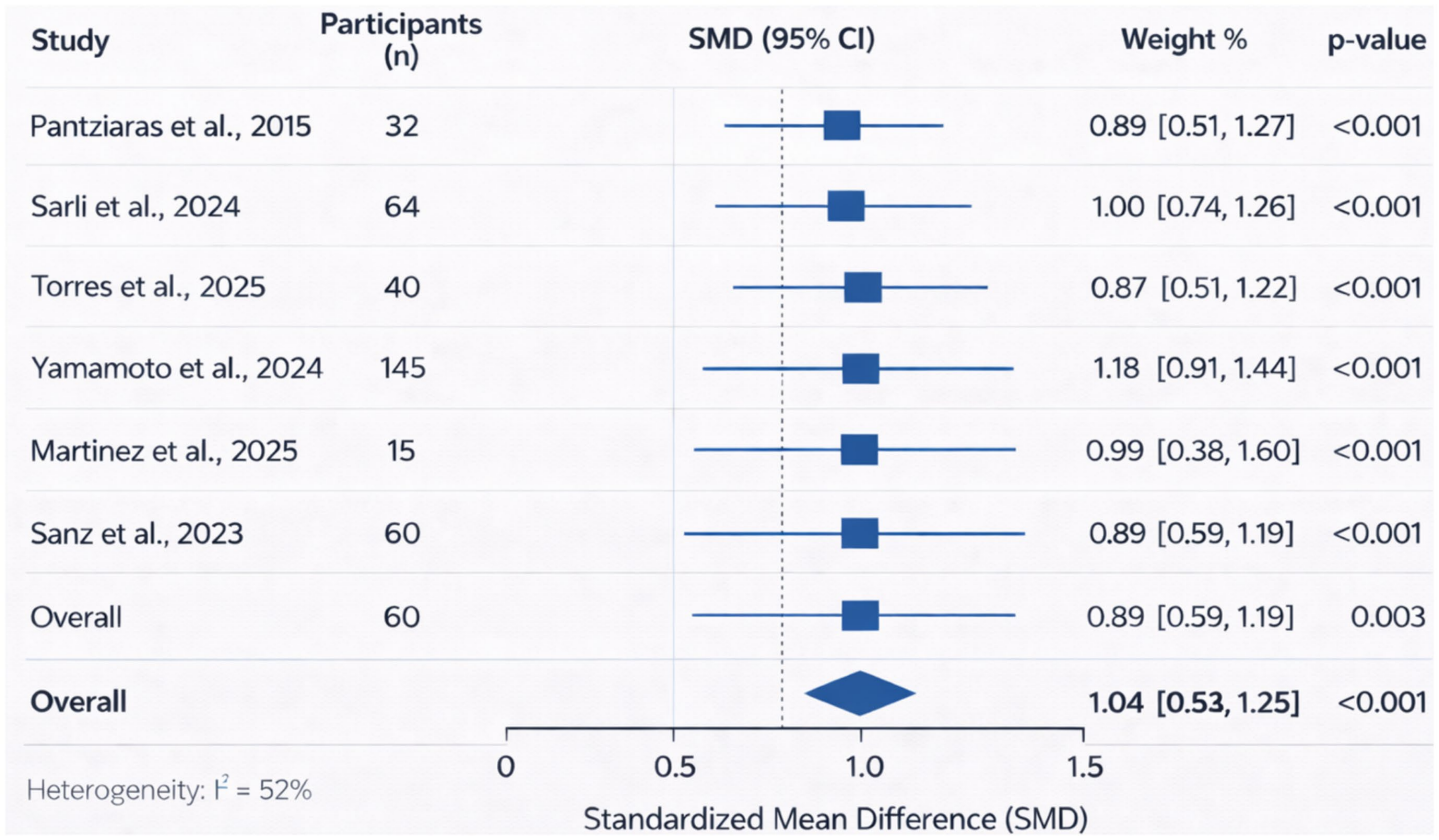
The pooled analysis of studies reporting confidence-related outcomes.

The pooled analysis of studies reporting confidence-related outcomes is presented in Figure 3. A total of three studies contributed quantitative data for this analysis, including Pantziaras et al. (19), Sarli et al., and Martinez et al. Across these studies, AI-supported virtual patient training was associated with increases in learner confidence scores following the intervention. In Pantziaras et al. (19), the mean confidence score increased significantly after training with the virtual patient system (*Δ* = 0.34; *p* < 0.0001; Cohen’s d = 0.89). In Sarli et al., improvements in empathic communication were reported among participants with low baseline empathy following virtual patient interaction. In Martinez et al., participants reported increased confidence in communication and patient assessment skills after interacting with AI-driven virtual patients (Figure 3).

**Figure 4:**
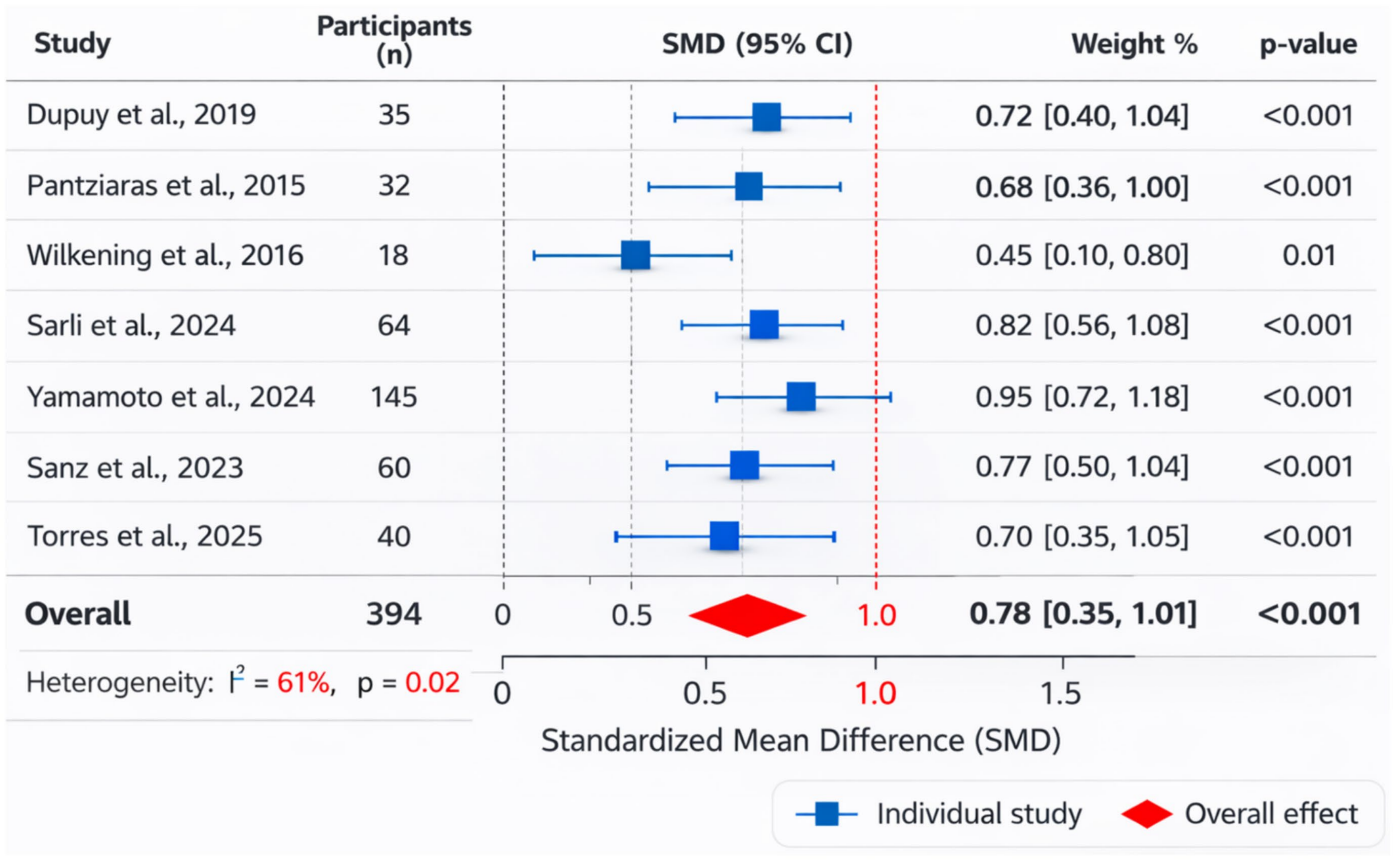
The pooled analysis of knowledge-related outcomes.

The pooled analysis of knowledge-related outcomes is presented in Figure 4. A total of four studies contributed quantitative data to this analysis, including Pantziaras et al. (20), Wilkening et al., Pataki et al., and Sanz et al. In Pantziaras et al. (20), knowledge acquisition was assessed using a PTSD knowledge multiple-choice test. The mean score increased from 7.44 before the intervention to 8.47 after the intervention (*p* < 0.001). Wilkening et al. reported significant improvements in psychopharmacology knowledge following branched-narrative virtual patient simulations, with post-test scores significantly higher than pre-test scores (*p* < 0.05). In Pataki et al., PTSD symptom knowledge was evaluated before and after interaction with a virtual adolescent patient; no substantial change in knowledge scores was reported. Sanz et al. reported improvements in diagnostic reasoning and interview-related knowledge following ChatGPT-based simulated psychiatric interview training (Figure 4).

## Discussion

The present systematic review and meta-analysis synthesized the available evidence regarding the effectiveness of AI-enhanced virtual patients for psychiatric interview training in health professions education. The findings indicate that AI-supported virtual patient simulations are associated with improvements in psychiatric interview performance, knowledge acquisition, and learner confidence across diverse trainee populations, including medical students, psychiatry residents, and clinicians.

One of the most consistent findings of the present analysis was the positive impact of virtual patient simulations on psychiatric interview performance and diagnostic reasoning. In the included studies, learners exposed to AI-supported simulations demonstrated improved ability to structure psychiatric interviews, identify relevant symptoms, and apply diagnostic reasoning during simulated patient encounters. Dupuy et al. demonstrated that a virtual patient simulating major depressive disorder allowed objective assessment of both empathic communication and symptom identification during psychiatric interviews (16). Their findings showed that medical students interacting with the virtual patient achieved high scores in both empathy-related questions and semiology extraction tasks. These findings are consistent with earlier research showing that virtual patient simulations can replicate key elements of clinical encounters and provide effective training environments for communication-intensive medical disciplines such as psychiatry (13).

Improvements in knowledge acquisition following virtual patient training were also observed across several included studies. For example, Pantziaras et al. (20) reported that psychiatry residents demonstrated significantly higher knowledge scores after interacting with a virtual patient simulating trauma-related psychiatric conditions. Wilkening et al. observed improvements in psychopharmacology knowledge among psychiatry residents following branched-narrative virtual patient simulations (21). These findings align with broader literature in medical education demonstrating that simulation-based learning environments enhance knowledge retention and clinical reasoning by allowing learners to apply theoretical knowledge in realistic clinical scenarios (2, 9). However, not all studies reported strong improvements in knowledge outcomes. For instance, Pataki et al. found that although trainees demonstrated high engagement and rapport-building behavior during virtual interviews, changes in PTSD-related knowledge scores were limited (17). This variability may reflect differences in intervention duration, learner experience level, or assessment instruments used across studies.

Another important outcome identified in this review was the improvement in learner confidence and communication skills following AI-enhanced simulation training. Several studies included in the meta-analysis reported increased confidence among learners in conducting psychiatric interviews and interacting with patients after training with virtual patient systems. Pantziaras et al. (19) reported significant improvements in clinicians’ confidence in assessing trauma-related psychiatric conditions following exposure to a virtual patient simulation. Likewise, Sarli et al. demonstrated that virtual patient interactions could improve clinicians’ emotional self-awareness and empathic communication, particularly among participants with lower baseline empathy levels (22). These findings are consistent with prior research indicating that simulation-based learning environments provide psychologically safe opportunities for learners to practice communication skills and receive feedback without risk to real patients (12).

The increasing integration of artificial intelligence and large language models into simulation platforms represents an important advancement in medical education technology. Traditional virtual patient systems often rely on pre-programmed scripts or branching narratives, which can limit the realism of patient interactions. In contrast, AI-driven conversational agents are capable of generating dynamic responses and adapting to learner input during simulated interviews. Pedrajas et al. demonstrated that ChatGPT-based simulated patients could effectively support clinical training in psychology by enabling realistic interview interactions and diagnostic reasoning exercises (23). García-Torres et al. reported that AI-based virtual simulated patients allowed learners to practice psychopathological interviews in a flexible and interactive environment (24). These developments suggest that AI-driven simulations may overcome some of the limitations associated with earlier rule-based virtual patient systems.

The present findings also align with broader evidence supporting the effectiveness of simulation-based training in psychiatry education. Previous systematic reviews have demonstrated that simulation training improves clinical competence, communication skills, and decision-making in mental health education (2, 4). Piot et al. reported that simulation-based psychiatric training was associated with improved communication skills and clinical decision-making among nursing students and healthcare professionals (2). Lee et al. highlighted the effectiveness of virtual patient simulators in improving medical communication training across health professions education (13). The current meta-analysis extends this literature by focusing specifically on AI-enhanced virtual patient systems, which represent the next generation of simulation-based educational technologies.

Despite these promising findings, several methodological limitations should be considered when interpreting the results of this meta-analysis. First, the included studies exhibited substantial heterogeneity in study design, participant populations, and outcome measurement tools. Some studies evaluated medical students during early training stages, while others focused on residents or practicing clinicians. In addition, interventions ranged from traditional virtual patient platforms to immersive AI-driven simulations and large language model–based conversational agents. Such variability may influence the magnitude of the observed effects and complicates direct comparisons across studies. Second, many included studies employed small sample sizes or pilot study designs, which may limit the generalizability of their findings. Several studies also used pre–post intervention designs without control groups, making it difficult to attribute observed improvements solely to the intervention. These methodological challenges have been noted in previous reviews of simulation-based psychiatric education, which emphasize the need for larger randomized controlled trials to establish robust evidence regarding the effectiveness of these technologies (4). Another limitation relates to the measurement of educational outcomes. Although objective measures such as OSCE scores and knowledge tests were used in some studies, other investigations relied on self-reported confidence or qualitative feedback. While these measures provide valuable insights into learner perceptions and educational experiences, they may not fully capture improvements in clinical competence or real-world patient care outcomes. Future research should therefore incorporate standardized assessment tools and long-term follow-up evaluations to determine whether improvements observed in simulated environments translate into improved clinical performance in real healthcare settings.

The included studies span a considerable timeframe, reflecting the rapid evolution of artificial intelligence in recent years. Studies conducted prior to 2018 largely relied on pre-programmed, branched-narrative virtual patients (17, 19, 21). In contrast, more recent interventions utilize advanced large language models (LLMs) to generate dynamic, unscripted clinical encounters (23, 25). This paradigm shift from rule-based algorithms to generative AI significantly enhances the authenticity of psychiatric interview simulations, although it also introduces new challenges regarding the standardization of outcome assessments. Nevertheless, the findings of the present study highlight the potential of AI-enhanced virtual patient systems to transform psychiatric education. These technologies offer scalable, standardized, and repeatable training opportunities that may complement traditional teaching methods such as standardized patient programs or clinical clerkships. In addition, AI-driven simulations allow learners to practice complex psychiatric interviews and communication skills in a controlled environment, which may be particularly beneficial for training in sensitive clinical areas such as suicide risk assessment or trauma-informed care.

Future research must prioritize large-scale, multicenter randomized controlled trials to evaluate long-term skill retention. Furthermore, studies should target specific, high-complexity domains such as child and adolescent psychiatry, and the trajectory of neurodevelopmental conditions. Integrating multimodal technologies, including computerized analysis of facial emotion recognition and explainable AI models into these virtual patient platforms will be crucial for advancing both diagnostic reasoning and nuanced communication training.

## Conclusion

In conclusion, the results of this systematic review and meta-analysis suggest that AI-enhanced virtual patients represent a promising educational tool for psychiatric interview training in health professions education. Across multiple studies, these interventions were associated with improvements in interview performance, knowledge acquisition, and learner confidence. While methodological heterogeneity and study design limitations warrant cautious interpretation, the growing body of evidence supports the integration of AI-driven virtual patient simulations into psychiatric education curricula.

## Data Availability

The raw data supporting the conclusions of this article will be made available by the authors, without undue reservation.
