# Correction: Effectiveness of AI-enhanced virtual patients for psychiatric interview training in health professions education: a meta-analysis

**DOI:** 10.3389/fmed.2026.1882263

**Published:** 2026-06-05

**Authors:** Senay Kilincel, Furkan Bulut, Pelin Goksel, Mirac Baris Usta, Tuba Mutluer, Oguzhan Kilincel

**Affiliations:** 1 Department of Child and Adolescent Psychiatry, School of Medicine, Istanbul Aydin University, Istanbul, Türkiye; 2 Private Practice, Psychotherapy Institute, Sakarya, Türkiye; 3 Department of Adult Psychiatry, School of Medicine, Ondokuz Mayis University, Samsun, Türkiye; 4 Department of Child and Adolescent Psychiatry, School of Medicine, Ondokuz Mayis University, Samsun, Türkiye; 5 Department of Child and Adolescent Psychiatry, Koc University, Istanbul, Türkiye; 6 Department of Child and Adolescent Psychiatry, School of Medicine, Akdeniz University, Istanbul, Türkiye; 7 Department of Child Development, Istanbul Gelisim University, Istanbul, Türkiye

**Keywords:** artificial intelligence, clinical communication skills, health professions education, medical education, meta-analysis, psychiatric interview training, simulation-based learning, virtual patients

Affiliation “Department of Child and Adolescent Psychiatry, School of Medicine, Akdeniz University, Istanbul, Türkiye”, was omitted for author Tuba Mutluer. This affiliation has now been added for author Tuba Mutluer.

The original version of this article has been updated.

